# Lymphatic filariasis in Luangwa District, South-East Zambia

**DOI:** 10.1186/1756-3305-6-299

**Published:** 2013-10-15

**Authors:** Sheila Tamara Shawa, Enala T Mwase, Erling M Pedersen, Paul E Simonsen

**Affiliations:** 1 Department of Paraclinical Studies, School of Veterinary Medicine, University of Zambia, Lusaka, Zambia; 2 Department of Veterinary Disease Biology, Faculty of Health and Medical Sciences, University of Copenhagen, Thorvaldsensvej 57, 1871 Frederiksberg C, Denmark

**Keywords:** Lymphatic filariasis, *Wuchereria bancrofti*, Circulating filarial antigens, Antibodies to Bm14, Microfilariae, Vector mosquitos, Human perception, Epidemiology, Zambia

## Abstract

**Background:**

Past case reports and recent data from LF mapping surveys indicate that LF occurs in Zambia, but no studies have been carried out to document its epidemiology and health implications. The present study assessed infection, disease, transmission and human perception aspects of LF in an endemic area of Luangwa District, South-East Zambia, as a background for planning and implementation of control.

**Methods:**

Two neighbouring rural communities were registered and a questionnaire survey undertaken. Clinical examination, and sampling of blood for circulating filarial antigens (CFA; marker of adult worm infection) and antibodies to Bm14 antigen (marker of exposure to transmission), were carried out during the daytime. Blood from CFA positive individuals was examined for microfilariae (mf) at night. Vector surveys were carried out in selected households, using light traps.

**Results:**

985 individuals aged ≥ 1 year were registered. The CFA prevalence increased with age from 1.2% in age group 1–14 years to 20.6% in age group 50+ years (overall 8.6%). *Wuchereria bancrofti* mf were identified in 10.9% of CFA positive individuals (corresponding to a community prevalence of 0.9%). Prevalence and intensity of Bm14 antibodies were much higher in individuals ≥ 30 years than in younger individuals (57.2 vs. 19.3%; 0.594 vs. 0.241 OD-values). Elephantiasis and hydrocele were well known clinical manifestations in the area, but only one case of hydrocele was detected in the study population. Identified potential vectors were *Anopheles funestus* and *An. gambiae.*

**Conclusion:**

The study confirmed that LF was endemic in the study communities, but infection and disease prevalence was low. Several indications, including a marked recent decline in CFA prevalence, suggest that transmission in the area is on the decrease, perhaps because of intensive application of malaria control measures targeting the *Anopheles* vectors. It is recommended that mass drug administration is initiated to accelerate this positive trend of decline in LF transmission in the area.

## Background

Many parts of Sub-Saharan Africa are known to be endemic for lymphatic filariasis (LF) [1], and detailed studies on the epidemiology and health implications of LF have been documented from several countries in this region. However, except for results from recent field surveys in Malawi [2-4] and a socio-environmental study on transmission risk in northern Mozambique [5], knowledge on LF infection, disease and transmission patterns in the central and southern region of Africa is limited. Available information from case reports and historic surveys indicate, though, that LF has a wider distribution in this part of Africa [6-9].

According to Buckley [10], the first cases of *Wuchereria bancrofti* microfilaraemia in Zambia were reported in 1938, and while undertaking a survey on human helminth infections in the northern and central part of the country, he recorded a few more cases. However, it was difficult to rule out the possibility of LF having been acquired elsewhere as the infected individuals were either not permanent residents in Zambia or had travelled to LF endemic neighbouring countries. He also indicated that there was evidence of LF infections being seen in Luangwa District (previously Feira District), and that clinical cases of elephantiasis - locally known as “Serenje leg” or “Feira Leg” - were seen in some districts of the country.

A small night blood survey carried out later in the Luangwa basin (Central Province) did not identify any microfilariae of *W. bancrofti *[11]. However, in 1975, Hira reported the first definite autochthonous case of *W. bancrofti* infection in a fisherman from Luangwa District who had never left Zambia [12,13]. Additional cases of LF infection, including autochthonous, were reported thereafter by Hira [13,14]. More recently, two cases of LF infection were reported from a 22 year old male and a 46 year old female from the southern and northern parts of the country, respectively [15,16]. In both cases microfilariae were identified in blood smears, and in the second case the female had elephantiasis suspected to be due to the *W. bancrofti* infection.

Despite these case reports from various parts of the country, very little is known about the epidemiology and health implications of LF in Zambia. Country-wide mapping of LF was undertaken between 2003 and 2011 and indicated that LF was widespread, although in most areas with relatively low prevalence [17]. As a partner in the Global Programme to Eliminate LF, Zambia is committed to eliminate LF as a public health problem. However, for planning and implementation of successful control, it is important to have a thorough knowledge about the local epidemiological characteristics of LF. The present study assessed infection, disease, transmission and human perception aspects of LF in an endemic area of Luangwa District, South-East Zambia.

## Methods

### Study sites

The study was carried out in Luangwa District (formerly Feira District) in South-East Zambia. The district is located in the Rift Valley, at the confluence of the Zambezi and Luangwa rivers at altitudes below 600 m above sea level. There are three main seasons: a cold season from May to August (temperature range 6-26°C), a hot season from September to October (17-35°C) and a rainy season from November to April (14-30°C). The main source of livelihood in the district is fishing on the rivers, production of reed mats and subsistence farming. The main cash crops are maize, groundnut, sweet potatoes, baobab fruit and pumpkins, and the common livestock are goats and chickens.

**Figure 1:**
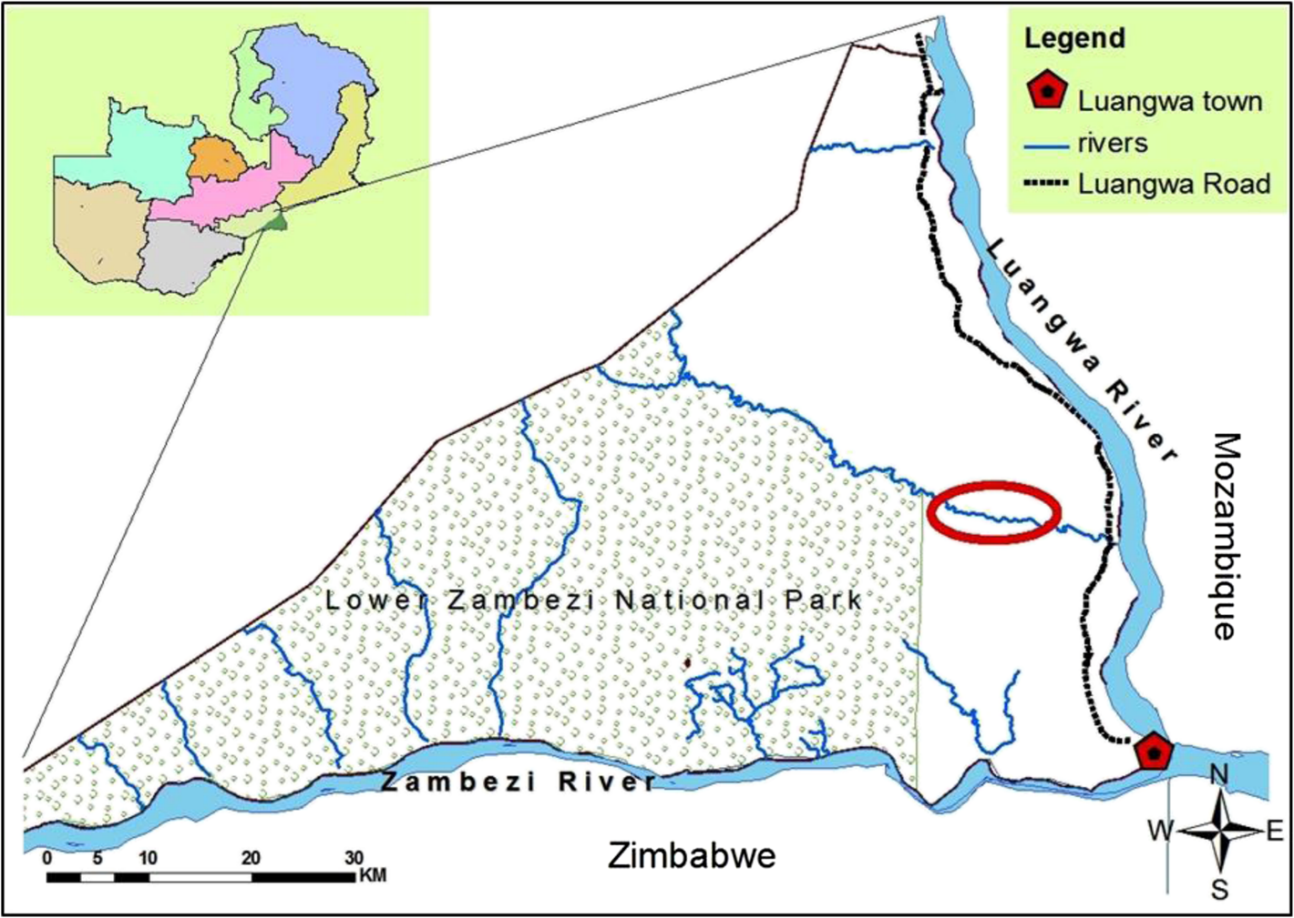
**Map showing the location of the study site in Luangwa District.** Red circle indicates location of households inhabited by the study population. Upper left corner shows the provinces in Zambia, and the location of Luangwa District in Lusaka Province.

A study site was identified based on findings from an LF mapping survey carried out in the area in 2003, when adults from the village of Janeiro, located approximately 35 km north of Luangwa town, were found to have a circulating filarial antigen (CFA) prevalence of 33.3% [17]. Janeiro (-15° 26′ 38S, 30° 21′ 31 E; altitude about 360 m) and its neighbouring village Yapite, with a combined population of approximately 1000 individuals, were selected for the present study (Figure 1). The two villages comprised a continuous stretch of scattered households along Rufunsa River (a tributary to Luangwa River) resembling each other closely in all ecological, cultural and household activity aspects, and were considered one study population for the purpose of the present study. Rufunsa River is a source of water for both villages for bathing, washing and other activities such as collection of reeds for making mats. The river also serves as a breeding site for mosquitos except during the dry season when the water dries up. Each of the villages had a health post.

### Study design

The study was cross-sectional. A house to house census was carried out in the study population in April/May 2011 during which the name, age and sex of individuals aged 1 year and above were registered. At the same time, a questionnaire survey was undertaken among individuals aged 15 years and above, who were present during the registration and accepted to take part. The survey captured information regarding presence of chronic LF disease (elephantiasis and hydrocele) in Luangwa District, attitudes and practices towards this disease, and possession and use of mosquito bed nets in the community. After the interview, information was given about LF and its transmission, treatment and prevention, and all household individuals aged 1 year and above were requested to come for examination at the health post.

Examination of blood for CFA (a marker of presence of adult *W. bancrofti* worms [18,19]) and collection of blood on filter paper for antibodies to Bm14 (a marker of exposure to transmission [20]) was carried out during the day at the health post. Individuals aged 15 years and above were examined clinically for chronic manifestations of LF. Individuals found positive for CFA were asked to come back to the health post between 21:30 and 01:00 hours for blood sampling for microfilaria investigation, due to the nocturnal periodicity of the microfilariae. Vector surveys were carried out in selected households after completion of the human surveys.

Ethical approval and research clearance for the study was provided by the University of Zambia Research and Ethics Committee. Permission was provided by the Ministry of Health, the Provincial and District Health office, the District Commissioner’s office, the local area chief and the village headmen. The objectives, requirements, benefits and duration of the study were explained to the villagers in the local language (Chikunda). LF leaflets and information sheets were also distributed. Verbal consent was obtained from all individuals before they participated in the examinations. Consent for individuals aged 15 years and below was given by parents/guardians. Children who were unwilling to take part in the study despite their parents/guardians consent were not included.

### Human examinations

Individuals aged 1 year and above were tested for CFA by use of Binax NOW Filariasis immunochromatographic test (ICT) cards (Inverness Medical Innovations, Maine, USA) according to manufacturer’s instructions. The results were read exactly 10 minutes after application of blood to the cards. A single band was interpreted as a negative result and two bands as a positive result.

Immediately following the CFA examination, additional 60 μl (6 drops of 10 μl) of finger prick blood was applied on filter paper blood collection discs (TropBio Pty Ltd, Townsville, Australia) according to instructions from the manufacturer. Each protrusion on the disc was saturated with blood from the same individual. The filter papers were left to dry thoroughly and then placed individually in small plastic bags and kept frozen at -20°C.

A clinician examined individuals aged 15 years and above for chronic manifestations of LF (elephantiasis and hydrocele). Manifestations were graded as previously described [21,22].

Individuals found positive for CFA were requested to meet the study team at the health post in the evening for microfilaria (mf) examination by use of the counting chamber technique [23]. Between 21:30 and 01.00 hours, 100 μl of blood was collected from a finger prick into a heparinised capillary tube and immediately transferred into specimen tubes with 1 ml of 3% acetic acid. The specimens were later examined in a counting chamber under a compound microscope, and the number of mf counted. Immediately after the first night blood sample, thick blood smears were prepared from the same individuals for accurate mf species identification and photography. The smears were de-haemoglobinized, fixed in methanol, stained with Giemsa and examined under a compound microscope.

### Vector surveys

Eight households with at least one individual found positive for CFA were selected for mosquito surveys that were carried out on 10 consecutive nights. Individuals sleeping in the collection room were provided with a rectangular un-impregnated mosquito bed net. A CDC miniature light trap (Model 512, John W. Hock Company, Florida, USA) was hung outside one of the nets, slightly above the head position of the person sleeping in the bed. The traps were turned on at 18.00 hours and off at 06.00 hours the following morning [24]. Trapped mosquitoes were transferred with an aspirator to paper cups covered with netting material. A cotton pad soaked in 10% glucose was placed on top of the netting material to allow mosquitos to feed while being transported. The cups were placed in a cool box and transferred to the field laboratory, where the mosquitoes were identified by morphological criteria.

The first vector survey was carried out in May 2011, but very few mosquitoes were caught. Two other similarly designed surveys were, therefore, carried out in August 2011 (with no mosquitoes caught) and March 2012.

### Laboratory examination for antibodies to Bm14

The frozen filter paper blood collection discs were brought to room temperature. One of the circular protrusions from each disc, containing approximately 10 μl of dried blood, was cut off and placed in an Eppendorf tube with 500 μl of extraction buffer and the sample was kept in a refrigerator at 4-6°C overnight for antibodies to elute.

The samples were thereafter analysed for IgG4 to the recombinant antigen Bm14 by enzyme linked immunosorbent assay (ELISA) by using the commercially available CELISA Bm14 kit (Cellabs Pty Ltd, Brookvale, Australia). Analyses were performed according to manufacturer’s instructions and as described elsewhere [25,26]. Negative and positive controls were prepared by adding 5 μl of the concentrated controls from the kit to 495 μl of extraction buffer in Eppendorf tubes. An ELISA reader (Thermo Scientific Multiskan FC spectrophotometer, Thermo Fischer Scientific Oy, Vantaa, Finland) was used to read the plates at dual wavelength of 450/620 nm, and the optical density (OD) was calculated by using the ELISA reader software. Each sample was tested in duplicate and the mean OD-value was recorded as the individuals’ result. Individuals with an OD-value ≥ 0.40 were considered antibody positive.

### Data analysis

Data were entered in Epi data (version 3.1), transported to Excel for cleaning and storage and finally exported to STATA (version 12) for analysis. Geometric mean intensities (GMIs) of Bm14 OD-values were calculated as antilog [(∑logx+1)/*n*]-1, with x being the OD-values and *n* the number of individuals included in the calculation. Categorical variables were compared by Chi-square (χ^2^) test, whereas continuous variables were compared by Student’s t-test. P-values less than 0.05 were considered statistically significant.

## Results

### Study population

A total of 985 individuals were registered in the two neighbouring study communities (Table 1). There were slightly more females (51.4%) than males (48.6%). The gender distribution in the four age groups was relatively equal with the exception of the oldest (50+ years), which had considerably more females than males. The study communities had 205 registered households, thus giving a mean number of individuals per household of 4.8 (range 1–11).

**Table 1:** Registered study population in Luangwa District and circulating filarial antigen (CFA) status among those examined

**Age group (years)**	**Study population**		**CFA**	
	**Total**	**Female: male ratio**	**No. examined**	**No. positive (%)**
1-14	473	0.89	248	3 (1.2)
15-29	241	1.25	138	15 (10.9)
30-49	156	0.95	92	15 (16.3)
50+	115	1.73	68	14 (20.6)
Total	985	1.06	546	47 (8.6)

### Circulating filarial antigens

**Figure 2:**
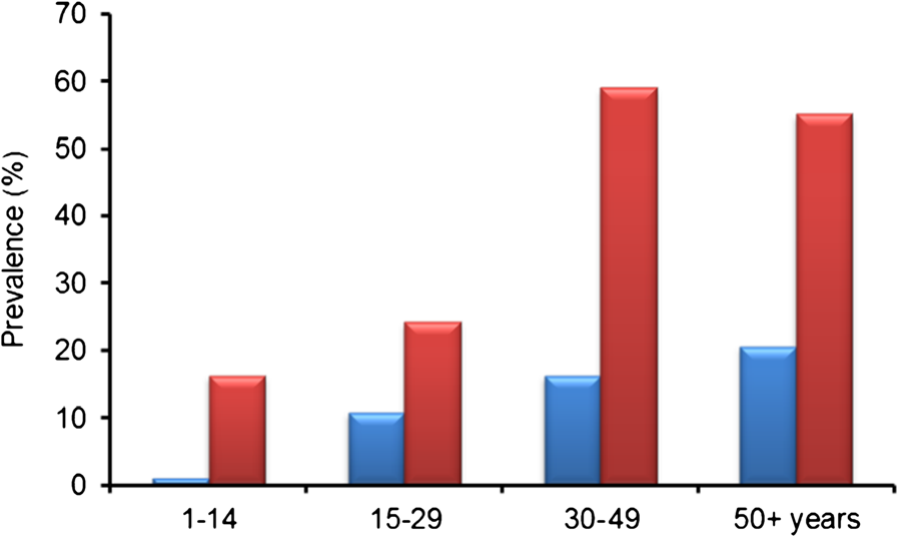
Prevalence of circulating filarial antigens (blue bars) and antibodies to Bm14 (red bars) in relation to age group in the study population in Luangwa District.

546 individuals (55.4% of those registered) were tested for CFA (Table 1). Of these, 47 were positive (overall CFA prevalence of 8.6%). The prevalence increased with age from 1.2% in the 1–14 years age group to 20.6% among those aged 50 years and more (Figure 2). The CFA prevalence in the 15+ years and 30+ years age groups was significantly higher than in individuals below these ages (14.8% vs. 1.2% and 18.1% vs. 4.7%; χ^2^ test, P < 0.001 for both tests). It was slightly but not significantly higher in males than in females (9.3%, n = 248 vs. 8.1%, n = 298; χ^2^ test, P > 0.05).

### Microfilariae

Night blood samples were collected from 46 of the 47 individuals found positive for CFA. Among these, 5 (10.9%) were positive for mf with the counting chamber technique. They were aged 27 (male), 47 (male), 49 (male), 50 (female) and 83 (male) years and had intensities of 1, 9, 11, 16 and 19 mf/100 μl of blood, respectively. On the assumption that the 5 mf positive individuals were the only mf positive individuals among the 546 CFA examined individuals, the mf prevalence among the CFA examined individuals was 0.9%, and among the 298 CFA examined individuals aged ≥ 15 years it was 1.7%.

**Figure 3:**
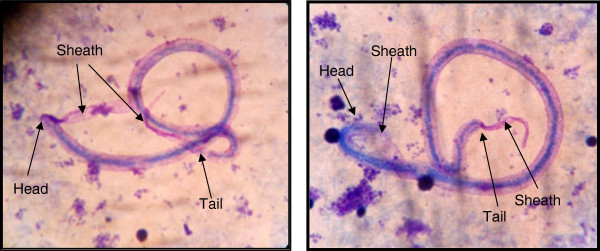
**Photographs of two microfilariae of *W. bancrofti *in thick blood smears stained with Giemsa.** The smears were prepared with blood from mf positive individuals from the study population in Luangwa District. Arrows point to characteristic features.

Thick blood smears were prepared from 25 of the 46 individuals examined with the counting chamber technique, and mf were found in smears from three of these. All three had also been found positive for mf using the counting chamber technique. The mf in the smears were identified as *W. bancrofti* on the basis of their graceful curves, pointed tails, lack of terminal nuclei and a clearly visible sheath protruding from both ends (Figure 3).

### Clinical findings

CFA examined individuals aged 15 years and above were also clinically examined for chronic manifestation of LF, namely 183 females and 115 males. No cases of elephantiasis were seen and only one male had hydrocele grade 2 (54 years, CFA negative).

### Antibodies to Bm14

The majority (538 or 98.5%) of CFA examined individuals were also examined for antibodies to Bm14 (Table 2). Of these, 164 were positive thus giving an overall Bm14 prevalence of 30.5%. The prevalence was lowest (16.4%) in the 1–14 years age group (Figure 2) and it was significantly higher in individuals aged 30+ years than in those below this age (57.2% vs. 19.3%, χ^2^ test, P < 0.001). It was slightly but not significantly higher in males than in females (34.0%, n = 247 vs. 27.5%, n = 291; χ^2^ test, P > 0.05).

**Table 2:** Antibodies to Bm14 among examined individuals in the study population in Luangwa District

**Age group (years)**	**No. examined**	**No. positive (%)**	GMI* among all	GMI* among positives
1-14	244	40 (16.4)	0.2157	0.7550
15-29	135	33 (24.4)	0.2874	0.9025
30-49	92	54 (58.7)	0.6167	1.0188
50+	67	37 (55.2)	0.5638	1.0068
Total	538	164 (30.5)	0.3361	0.9253

*) Geometric mean intensity, in OD-values.

When analysed for the same 538 individuals, the GMI of antibodies to Bm14 in general followed the same patterns as the prevalence. Thus, the lowest GMI was seen in the youngest age group (Table 2), and it was significantly higher in those aged 30+ years than in those below this age (0.5942 vs.0.2407 OD-values; t-test, P < 0.001). When analysed for the 164 Bm14 positive individuals, a similar pattern of GMIs was seen (Table 2), although as expected the levels were higher than for GMIs among all examined. The GMI among positive individuals was significantly higher in those aged 30+ years compared to those below this age (1.0139 vs. 0.8202 OD-values; t-test, P = 0.017).

### Relationship between CFA, antibodies to Bm14, mf and clinical manifestations

The relationship between CFA status and antibodies to Bm14 was analysed for the 538 individuals examined for both of these markers (Table 3). The prevalence of antibodies to Bm14 was significantly higher among CFA positive than among CFA negative individuals (57.4% vs. 27.9%; χ^2^ test, P < 0.001). The GMI of OD-values was also significantly higher among CFA positive individuals than among those negative for CFA, both when calculated on the basis of all examined (0.6989 vs. 0.3143 OD-values; t-test, P < 0.001) and of Bm14 positives only (1.2628 vs. 0.8649 OD-values; t-test, P < 0.001).

**Table 3:** Relationship between circulating filarial antigen (CFA) status and antibodies to Bm14 in individuals examined for both markers in the study population in Luangwa District

**CFA status**	**No. examined**	**No. Bm14 positive (%)**	GMI* of Bm14 among all	GMI* of Bm14 among positives
+	47	27 (57.4)	0.6989	1.2628
-	491	137 (27.9)	0.3143	0.8649
All	538	164	0.3361	0.9253

*) Geometric mean intensity, in OD-values.

All five individuals with mf were positive for CFA, but only three of these were positive for antibodies to Bm14. The single male with hydrocele was positive for antibodies to Bm14 but negative for CFA and mf.

### Questionnaire survey

262 individuals aged 15 years and above (51.2% of those registered) took part in the questionnaire surveys (Table 4). The majority indicated they had seen cases of elephantiasis (79%; locally called Nsakasa) and hydrocele (75.5%; locally called Nchofu, Nthumbo or Pholo) in Luangwa district. A significantly higher percentage of individuals aged 30+ years compared to those below this age had seen cases of elephantiasis (83.3% vs. 72.0%; χ^2^test, P = 0.029) and hydrocele (80.2% vs. 67.7%; χ^2^test, P = 0.022). Despite the high level of recognition of these manifestations, only 29.0% and 27.1% indicated that elephantiasis and hydrocele, respectively, was acquired from mosquito bites. Individuals with elephantiasis and hydrocele were generally indicated to be perceived as everyone else (50% and 49.6%, respectively) or with compassion (33.2% and 33.9%, respectively).

**Table 4:** Responses from questionnaire-based assessment of the occurrence and naming of elephantiasis and hydrocele in the study population in Luangwa District

**Questions and responses**	**No. responses (%)**
Is this condition (elephantiasis)* found in Luangwa District? (n=262)	
Yes	207 (79.0)
No	55 (21.0)
Is this condition (hydrocele)* found in Luangwa District? (n=261)	
Yes	197 (75.5)
No	64 (24.5)
*What is elephantiasis called in your local language? (n=262)*	
Nsakasa	209 (79.8)
Other	12 (4.5)
Don’t know	41 (15.7)
*What is hydrocele called in your local language? (n=262)*	
Nchofu	56 (21.4)
Ntumbo	37 (14.1)
Pholo	19 (7.2)
Other	19 (7.3)
Don’t know	131 (50.0)
*How does one get elephantiasis? (n=262)*	
Mosquito bite	76 (29.0)
Witchcraft	45 (17.2)
Walking barefoot	25 (9.5)
Sexually transmitted	19 (7.3)
Other	12 (4.6)
Don’t know	85 (32.4)
*How does one get hydrocele? (n=262)*	
Mosquito bite	71 (27.1)
Witchcraft	30 (11.5)
Sexually transmitted	30 (11.5)
Inherited	18 (6.9)
Walking barefoot	13 (4.9)
Other	25 (9.5)
Don’t know	75 (28.5)
*How are individuals with elephantiasis perceived? (n=262)*	
Same as everyone	131 (50.0)
Compassion	87 (33.2)
Other	17 (6.5)
Don’t know	27 (10.3)
*How are individuals with hydrocele perceived? (n=262)*	
Same as everyone	130 (49.6)
Compassion	89 (33.9)
Other	14 (5.4)
Don’t know	29 (11.1)

n indicates total number of individuals responding.

*) Photograph of manifestation shown.

62.2% of the respondents indicated they had a mosquito bed net at home. Possession of nets was significantly more common among respondents aged 30+ years compared to those below this age (68.6% vs. 54.5%; χ^2^test, P *=* 0.023). 69.9% of those who possessed bed nets reported using them always, 25.2% sometimes and 4.9% rarely.

### Vector surveys

During the three vector surveys a total of 940 mosquitoes were caught, namely 123 (13.1% of total) during May 2011, 0 during August 2011 and 817 (86.9% of total) during March 2012 (Table 5). Among the three well known vectors of LF in Sub-Saharan Africa, *An. gambiae* (3.9% of total) and *An. funestus* (28.6% of total) were caught, but no *Cx. quinquefasciatus*. Among the potential vectors, *An. funestus* was much more common than *An. gambiae* (87.9% vs. 12.1%). Mosquitoes were not dissected for filarial infection, as the low mf prevalence in the humans population made it unlikely to find infected specimens.

**Table 5:** Mosquitoes caught using CDC light traps during the three surveys in the study community in Luangwa District

**Survey period**	**No. (%) mosquitoes caught**						**Total no. caught**
	***Anopheles gambiae***	***Anopheles funestus***	**Other anophelines**	***Culex quinquefasciatus***	***Aedes spp.***	**Other culicines**	
May 2011	4 (3.3)	63 (51.2)	49 (39.8)	0 (0.0)	0 (0.0)	7 (5.7)	123
August 2011	0 (-)	0 (-)	0 (-)	0 (-)	0 (-)	0 (-)	0
March 2012	33 (4.0)	206 (25.2)	54 (6.6)	0 (0.0)	0 (0.0)	524 (64.1)	817
All	37 (3.9)	269 (28.6)	103 (11.0)	0 (0.0)	0 (0.0)	531 (56.5)	940

## Discussion

As a partner in the Global Programme to Eliminate LF, Zambia is committed to national elimination of LF as a public health problem. Knowledge about the distribution, prevalence and other epidemiological characteristics of LF infection and disease is important for this endeavour to become successful. Country wide mapping commenced in 2003 and was completed in 2011 [17]. 78% of the numerous survey sites throughout the country were positive for CFA, with prevalences ranging from 1% to more than 50%. However, in general, prevalences were low and only at a few sites greater than 15%. Luangwa was among the districts with higher CFA prevalences, namely 25.0%, 33.0% and 36.3% at the three survey sites, and was therefore selected for further studies on the epidemiological characteristics of LF infection and disease.

The study was undertaken in the two neighbouring villages of Janeiro and Yapite. Households were sparsely distributed with most of them located in close proximity to the river. Some individuals including school children had to cross the river on a daily basis for school or other activities. Among the registered individuals the female: male ratio was almost equal, although the 50+ years age group comprised mainly of elderly women who were widowed. Only about half of the registered population was examined for CFA. Many individuals did not agree to have their blood drawn for religious reasons, others were at their temporal homes located near the fields harvesting their crops, and some refused to participate due to fear of being tested for HIV.

Detection of CFA by use of ICT test cards was used in the initial screening of individuals for LF. This diagnostic test is sensitive and specific, can be used at any time of day and allows for large numbers of individuals to be screened in a short period of time [18,19]. The prevalence of CFA was relatively low in young individuals and increased with age, as also seen in other endemic areas [27,28]. However, the overall CFA prevalence in the study population was low (14.8% among those aged ≥ 15 years), especially when considering that the prevalence reported from the same community and age group during the mapping survey eight years previously was 33.3%. The marked decrease in this short period of time suggests that a recent dramatic decline in transmission had taken place. This was supported by the trend seen in CFA status among 46 re-identified community individuals who had also been examined during the mapping survey in 2003. Among 17 CFA positives in 2003, only 5 were still positive in 2011 (71.6% had lost infection), while among 29 CFA negatives in 2003, only one was positive in 2011 (3.4% gain of infection). It is likely that in those who had reverted from infection positive to negative the *W. bancrofti* worms had died naturally and that no re-infection had taken place due to the low level of transmission. There were no clear gender differences in CFA prevalence, as often observed in other surveys, perhaps due to the generally low prevalence in the study community.

Many of the CFA positive individuals were negative for antibodies to Bm14 and vice versa, thus supporting the view that the Bm14 antibodies are not induced by the presence of adult worms but by exposure to infection [20,29]. Similar to CFA, the prevalence of antibodies to Bm14 also increased with increasing age. It was, however, higher than the CFA prevalence, both overall and in the individual age groups. However, whereas the increase in CFA prevalence was gradual, there was a rather abrupt increase in Bm14 prevalence, and also of Bm14 intensities, around the age of 30 years. The much higher prevalence and intensity of Bm14 in the older population might be due to differences in lifestyle, giving rise to marked differences in exposure between young and old individuals. It is also possible that it could be a reflection of a decline that has occurred in LF transmission in the more distant past, and that the older age groups had been much more exposed to filarial infection when they were young, and perhaps still sustained the Bm14 antibodies in their circulation. The significantly higher prevalence and intensity of Bm14 among the CFA positives than among CFA negatives suggests that CFA positive individuals had a higher chance of being exposed to infection than CFA negative individuals. This may not be surprising as the CFA positive group includes the mf positive individuals who supply infection to the vector mosquitoes and therefore keep the transmission going in their surroundings.

Mf examinations were undertaken in CFA positive individuals only, as CFA is a marker for the adult worms which produce the mf. The prevalence of microfilaraemia was low in the study population, and the youngest individual with mf was 27 years old. The mf prevalence is generally lower than the CFA prevalence, especially in low endemicity communities, where also the first cases of mf are usually seen at an older age than in high endemicity communities [28]. Previous reports have noted cases of another filarial species, *Mansonella perstans*, in Zambia [11,14]. However, in the present study none of the characteristic small *M. perstans* mf were detected with the counting chamber technique, and the mf in the blood smears were clearly identified as *W. bancrofti* on the basis of their morphological features.

The burden of chronic clinical LF disease was low in the study population, probably because of the general low level of endemicity [28]. This is in contrast to previous reports, which indicated that elephantiasis (Feira leg) and hydrocele were common in the area [10,11,13]. It might be speculated that a decrease in LF transmission in the past could have led to a reduction in the chronic disease burden. The questionnaire surveys revealed that a significantly higher proportion of individuals aged ≥ 30 years had seen cases of elephantiasis and hydrocele in the area compared to those who were younger, thus being another indication that LF may have been more common in the past. Officers from Luangwa District Health Office and the local Katondwe Mission Hospital informed the study team that hydroceles were still common and that hydrocelectomies were frequently carried out at the hospital. However, many individuals from nearby areas in Zimbabwe and Mozambique, where LF is also endemic [8,30], seek medical treatment from the mission hospital and it is likely that at least some of the hydrocele cases they report are from neighbouring countries. Despite the high percentages of questionnaire respondents who indicated to have seen cases of elephantiasis and hydrocele in the district, very few had knowledge on how these conditions were acquired; common responses included witchcraft, sexual transmission and walking barefoot (it is unlikely that the later referred to podoconiosis, a condition of endemic non-filarial elephantiasis induced by mineral particles from volcanic soils [31], as Luangwa is a lowland area with mainly alluvial soils). The responses underscored the importance of sensitization of the community populations before implementation of MDA, to ensure maximum co-operation and compliance [32].

Studies on mosquitoes and their role in malaria transmission have been carried out in some parts of Zambia [33-35] including Luangwa District [36,37], but there have been no documented studies on vectors responsible for LF transmission in the country. During the first two vector surveys of the present study very few or no mosquitos were caught, probably due to low temperatures. Several species of mosquitoes were caught during the third survey, including *An. funestus* and *An. gambiae*, which are the two main LF vectors in Sub-Saharan Africa [38], including neighboring Malawi [39]. It is likely that these two species are also the main vectors of LF in Luangwa District. In agreement with other studies carried out in the same district on malaria vectors [36,37], *An. funestus* was much more common than *An. gambiae*, and this species is probably the principal LF vector in the area. Dark-pigmented *Cx. quinquefasciatus* have been reported from the Southern Province of Zambia [40], but no potential vectors of this species were caught in Luangwa District.

The questionnaire survey revealed that the majority of respondents possessed bed nets, and that most of those owning them also frequently used them. Similar high usage of bed nets has been reported previously from Luangwa District and from other parts of Zambia [41-43]. Zambia has a well-established malaria control programme, with an active integrated vector control part involving distribution of bed nets and indoor residual spraying, which has rapidly expanded the geographical coverage since 2004 [43-45]. As the vectors for malaria and LF are the same, it is likely that these malaria vector control measures have simultaneously reduced exposure to filarial parasites and could be the major course for the observed current low prevalence of LF in the study population. Vector control measures have similarly been reported to be responsible for a decline in LF prevalence in endemic communities elsewhere [46,47], and have been recommended as important additional measures for LF control [38,43].

## Conclusion

The present study confirmed that LF was endemic in Luangwa District, but the burden of infection and disease was low. There were several indications that LF transmission in the area had decreased, in particular the low CFA prevalence when compared to that observed during the mapping survey eight years previously. This was likely due to a decrease in LF transmission following intensive application of malaria vector control in recent years. A very high Bm14 level in the older individuals, and a low burden of chronic LF disease compared to what was reported previously, could indicate that also a more long-term decrease in LF transmission had taken place. It is recommended that MDA is initiated to support and accelerate this positive trend of decline in LF transmission. As the neighbouring countries are also endemic for LF, a co-ordinated cross-country approach would be most fruitful for the efforts to eliminate LF as a public health problem in the area.

## Competing interests

The authors declare that they have no competing interests.

## Authors’ contributions

STS, ETM, EMP and PES conceived and designed the study. STS collected the field data under the supervision of ETM, EMP and PES. STS performed the laboratory tests, analysed the data and drafted the paper under the supervision of PES. All authors read and approved the final version of the manuscript.

## References

[B1] Hotez PJ Kamath A Neglected tropical diseases in Sub-Saharan Africa: review of their prevalence, distribution, and disease burden PLoS Negl Trop Dis 2009 3 e412 10.1371/journal.pntd.0000412 19707588 PMC2727001

[B2] Ngwira BMM Jabu CH Kanyongoloka H Mponda M Crampin AC Branson K Alexender NDE Fine PEM Lymphatic filariasis in the Karonga district of Northern Malawi: a prevalence survey Ann Trop Med Parasitol 2002 96 137 144 10.1179/0003498302125000411 12080974

[B3] Ngwira BMM Tambala P Perez AM Bowie C Molyneux DH The geographical distribution of lymphatic filariasis infection in Malawi Filaria J 2007 6 12 10.1186/1475-2883-6-12 18047646 PMC2233609

[B4] Nielsen NO Makaula P Nyakuipa D Bloch P Nyasulu Y Simonsen PE Lymphatic filariasis in lower Shire, Southern Malawi Trans R Soc Trop Med Hyg 2002 96 133 138 10.1016/S0035-9203(02)90279-8 12055799

[B5] Manhenje I Galan-Puchades MT Fuentes MV Socio-environmental variables and transmission risk of lymphatic filariasis in central and northern Mozambique Geospat Hlth 2013 7 391 398 10.4081/gh.2013.96 23733300

[B6] Sasa M Human filariasis: A global survey of epidemiology and control 1976 University of Tokyo Press

[B7] Hawking F The distribution of human filariasis throughout the World part III. Africa Trop Dis Bull 1977 74 649 679 919037

[B8] Fujita K ODA T Tsukidate S Mori A Ueda M Kurokawa K Preliminary report on human filariasis in Mozambique, East Africa Trop Med 1985 27 83 91

[B9] Kelly-Hope LA Thomas BC Bockerie MJ Molyneux DH Lymphatic filariasis in the Democratic Republic of Congo; micro-stratification overlap mapping (MOM) as a prerequisite for control and surveillance Parasit Vectors 2011 4 178 10.1186/1756-3305-4-178 21923949 PMC3183006

[B10] Buckley JJC A helminthological survey in Northern Rhodesia J Helminthol 1946 21 111 174 10.1017/S0022149X00032041

[B11] Barclay R Filariasis in Luangwa basin Med J Zambia 1971 5 201 203

[B12] Hira PR Bancroftian filariasis in Zambia Ann Trop Med Parasitol 1975 69 521 522

[B13] Hira PR Bancroftian filariasis: an autochthonous case in Zambia Med J Zambia 1976 10 160 163 1052106

[B14] Hira PR *Wuchereria bancrofti*: the staining of the microfilarial sheath in giemsa and haematoxylin for diagnosis Med J Zambia 1977 11 93 96 919785

[B15] Kaile T Filariasis presenting as pyrexia of unknown origin in a young Zambian adult: a case report Zambian J Med Health Sci 1998 2 2 3

[B16] Matondo AS Lungu AG Lymphatic filariasis in a Zambian woman: a case report Zambian J Med Health Sci 1998 2 55 56

[B17] Mwase ET Stensgaard AS Msakashalo-Senkwe M Mubila L Mwansa J Songolo P Shawa ST Simonsen PE Mapping the geographical distribution of lymphatic filariasis in Zambia PLoS Negl Trop Dis In press 10.1371/journal.pntd.0002714 PMC3930513 24587466

[B18] Weil GJ Lammie PJ Weiss N The ICT filariasis test: a rapid-format antigen test for diagnosis of bancroftian filariasis Parasitol Today 1997 13 401 404 10.1016/S0169-4758(97)01130-7 15275155

[B19] Weil GJ Ramzy RMR Diagnostic tools for filariasis elimination programs Trends Parasitol 2006 23 2 10.1016/j.pt.2006.12.001 17174604

[B20] Tisch DJ Bockarie MJ Dimber Z Kiniboro B Tarongka N Hazlett FE Kastens W Alpers MP Kuzura JW Mass drug administration trail to eliminate lymphatic filariasis in Papua New Guinea: changes in microfilaraemia, filarial antigen, and Bm14 antibody after cessation Am J Trop Med Hyg 2008 78 289 293 18256431 PMC2590750

[B21] Estambale BBA Simonsen PE Knight R Bwayo JJ Bancroftian filariasis in Kwale District of Kenya. I. clinical and parasitological survey in an endemic community Ann Trop Med Parasitol 1994 88 145 151 8067810 10.1080/00034983.1994.11812852

[B22] Meyrowitsch DW Simonsen PE Makunde WH Bancroftian filariasis: analysis of infection and disease in five endemic communities of north-eastern Tanzania Ann Trop Med Parasitol 1995 89 653 663 8745940 10.1080/00034983.1995.11812999

[B23] McMahon JE Marshall TF Vaughan JP Abaru DE Bancroftian filariasis: a comparison of microfilariae counting techniques using counting chamber standard slide and membrane (nuclepore) filtration Ann Trop Med Hyg 1979 73 457 464 10.1080/00034983.1979.11687285 393190

[B24] Rwegoshora RT Pedersen EM Mukoko DA Meyrowitsch DW Masese N Malecela–Lazaro MN Ouma JH Michael E Simonsen PE Bancroftian filariasis: patterns of vector abundance and transmission in two east African communities with different levels of endemicity Ann Trop Med Parasitol 2005 99 253 265 10.1179/136485905X29675 15829135

[B25] Joseph HM Melrose W Applicacility of the filter paper technique for detection of antifilarial IgG4 antibodies using the Bm14 filariasis CELISA J Parasitol Res 2010 Article ID 594687 10.1155/2010/594687 PMC2911587 20700424

[B26] Weil GJ Curtis KC Fischer PU Won KY Lammie PJ Joseph H Melrose WD Brattig NW A multicentre evaluation of a new antibody test kit for lymphatic filariasis employing recombinant *Brugia malayi* antigen Bm-14 Acta Trop 2011 120s s19 s22 20430004 10.1016/j.actatropica.2010.04.010 PMC2935504

[B27] Onapa AW Simonsen PE Pedersen EM Okelle DO Lymphatic filariasis in Uganda: baseline investigations in Lira, Soroti and Katawi districts Trans R Soc Trop Med Hyg 2001 95 161 167 10.1016/S0035-9203(01)90145-2 11355548

[B28] Simonsen PE Meyrowitsch DW Jaoko WG Malecela MN Mukoko D Pedersen EM Ouma JH Rwegoshora RT Masese N Magnussen P Estambale BBA Micheal E Bancroftian filariasis infection, disease, and specific antibody response patterns in a high and a low endemicity community in East Africa Am J Trop Med Hyg 2002 66 550 559 12201589 10.4269/ajtmh.2002.66.550

[B29] Joseph H Maiava F Naseri T Silva U Lammie P Melrose W Epidemiological assessment of continuing transmission of lymphatic filariasis in Samoa Ann Trop Med Parasitol 2011 105 567 578 10.1179/2047773211Y.0000000008 22325816 PMC4089807

[B30] Roberts CJ Whitehall J Gelfand M *W. bancrofti* in Kenyemba area Cent Afr J Med 1973 19 13 14 4712840

[B31] Davey G Tekola F Newport MJ Podoconiosis: non-infectious geochemical elephantiasis Trans R Soc Trop Med Hyg 2007 101 1175 1180 10.1016/j.trstmh.2007.08.013 17976670

[B32] Parker M Allen T Will mass drug administration eliminate lymphatic filariasis? Evidence from northern coastal Tanzania J Biosoc Sci 2013 45 517 545 10.1017/S0021932012000466 23014581 PMC3666211

[B33] Kent RJ The mosquitoes of Macha, Zambia 2006 The John Hopkins Malaria Research Institute http://www.jhsph.edu/malaria

[B34] Kent RJ Thuma PE Mrakurva S Norris DE Seasonality, blood feeding behaviour, and transmission of *Plasmodium falciparum* by *Anopheles arabiensis* after an extended draught in southern Zambia Am J Trop Med Hyg 2007 76 267 274 17297034 PMC4152308

[B35] Fornadel CM Norris LC Glass GE Norris DE Analysis of *Anopheles arabiensis* blood feeding behaviour in southern Zambia during the two years after introduction of insecticide-treated bed nets Am J Trop Med Hyg 2010 84 848 853 20889878 10.4269/ajtmh.2010.10-0242 PMC2946755

[B36] Seyoum A Sikaala CH Canda J Chinula D Ntamatungiro AJ Hawela M Miller JM Russell TL Briet OJT Killeen G Human exposure to anopheline mosquitoes occurs primarily indoors, even for users of insecticide-treated nets in Luangwa Valley, South-east Zambia Parasit Vectors 2012 5 101 10.1186/1756-3305-5-101 22647493 PMC3432592

[B37] Sikaala CH Killeen GF Chanda J Chinula D Miller JM Rusell TL Seyoum A Evalutaion of alternative mosquito sampling methods for malaria vectors in lowland South-East Zambia Parasit Vectors 2013 6 91 10.1186/1756-3305-6-91 23570257 PMC3639086

[B38] Bockarie MJ Pedersen EM White GB Micheal E Role of vector control in the global program to eliminate lymphatic filariasis Ann Rev Entomol 2009 54 469 487 10.1146/annurev.ento.54.110807.090626 18798707

[B39] Merelo-Lobo RA McCall PJ Perez MA Spiers AA Mzilahowa T Ngwire B Molyneux DH Donelly MJ Identification of the vectors of lymphatic filariasis in the Lower Shire Valley, southern Malawi Trans R Soc Trop Med Hyg 2003 97 299 301 10.1016/S0035-9203(03)90149-0 15228246

[B40] Kent RJ Mrakurva S Hamapumbu H Norris DE Recognition of a novel melanotic mutant in a field population of *Culex pipiens quinquefasciatus* in southern Zambia J Am Mosq Contr Assoc 2007 23 71 75 10.2987/8756-971X(2007)23[71:ROANMM]2.0.CO;2 PMC4152315 17536371

[B41] Chizema-Kawesha E Miller JM Steketee RW Mukonka VM Mukuka C Mohamed AD Miti SK Campbell CC Scaling up malaria control in Zambia: Progress and impact 2005–2008 Am J Trop Med Hyg 2010 83 480 488 10.4269/ajtmh.2010.10-0035 20810807 PMC2929038

[B42] Larsen DA Keating J Miller J Bennett A Changufu C Katebe C Eisele TP Barriers to insecticide-treated mosquito net possession 2 years after a mass free distribution campaign in Luangwa District Zambia PloS One 2010 5 e13129 10.1371/journal.pone.0013129 21085711 PMC2978084

[B43] Kelly-Hope LA Molyneux DH Bockarie MJ Can malaria vector control accelerate the interruption of lymphatic filariasis transmission in Africa; capturing a window of opportunity? Parasit Vectors 2013 6 39 10.1186/1756-3305-6-39 23433078 PMC3599698

[B44] Chanda E Masaninga F Coleman M Sikaal C Katebe C MacDonald M Baboo KS Govere J Manga L Integrated vector management: the Zambian experience Malaria J 2008 7 164 10.1186/1475-2875-7-164 PMC2551620 18752658

[B45] Chanda E Hemingway J Kleinschmidt I Rehman AM Phiri FN Coetzer S Mthembu D Shinondo CJ Chizema-Kawesha E Kamuliwo M Mukonka V Baboo KS Coleman M Insecticide resistance and the future of malaria control in Zambia PLoS One 2011 6 e24336 10.1371/journal.pone.0024336 21915314 PMC3167838

[B46] Webber RH The natural decline of *Wuchereria bancrofti* infection in a vector control situation in the Solomon Islands Trans R Soc Trop Med Hyg 1977 71 396 400 10.1016/0035-9203(77)90037-2 595094

[B47] Njenga SM Mwandawiro CS Wamae CN Mukoko DA Omar AA Shimada M Bockarie MJ Molyneux DH Sustained reduction in prevalence of lymphatic filariasis infection in spite of missed rounds of mass drug administration in an area under mosquito nets for malaria control Parasit Vectors 2011 4 90 10.1186/1756-3305-4-90 21612649 PMC3125382

